# Inotropic Action of the Puberty Hormone Kisspeptin in Rat, Mouse and Human: Cardiovascular Distribution and Characteristics of the Kisspeptin Receptor

**DOI:** 10.1371/journal.pone.0027601

**Published:** 2011-11-22

**Authors:** Janet J. Maguire, Helen R. Kirby, Emma J. Mead, Rhoda E. Kuc, Xavier d'Anglemont de Tassigny, William H. Colledge, Anthony P. Davenport

**Affiliations:** 1 Clinical Pharmacology Unit, University of Cambridge, Cambridge, United Kingdom; 2 Department of Physiology, Development and Neuroscience, University of Cambridge, Cambridge, United Kingdom; University of Padova, Medical School, Italy

## Abstract

Kisspeptins, the ligands of the kisspeptin receptor known for its roles in reproduction and cancer, are also vasoconstrictor peptides in atherosclerosis-prone human aorta and coronary artery. The aim of this study was to further investigate the cardiovascular localisation and function of the kisspeptins and their receptor in human compared to rat and mouse heart. Immunohistochemistry and radioligand binding techniques were employed to investigate kisspeptin receptor localisation, density and pharmacological characteristics in cardiac tissues from all three species. Radioimmunoassay was used to detect kisspeptin peptide levels in human normal heart and to identify any pathological changes in myocardium from patients transplanted for cardiomyopathy or ischaemic heart disease. The cardiac function of kisspeptin receptor was studied in isolated human, rat and mouse paced atria, with a role for the receptor confirmed using mice with targeted disruption of *Kiss1r*. The data demonstrated that kisspeptin receptor-like immunoreactivity localised to endothelial and smooth muscle cells of intramyocardial blood vessels and to myocytes in human and rodent tissue. [^125^I]KP-14 bound saturably, with subnanomolar affinity to human and rodent myocardium (K_D_ = 0.12 nM, human; K_D_ = 0.44 nM, rat). Positive inotropic effects of kisspeptin were observed in rat, human and mouse. No response was observed in mice with targeted disruption of *Kiss1r*. In human heart a decrease in cardiac kisspeptin level was detected in ischaemic heart disease. Kisspeptin and its receptor are expressed in the human, rat and mouse heart and kisspeptins possess potent positive inotropic activity. The cardiovascular actions of the kisspeptins may contribute to the role of these peptides in pregnancy but the consequences of receptor activation must be considered if kisspeptin receptor agonists are developed for use in the treatment of reproductive disorders or cancer.

## Introduction

The pairing of the kisspeptin receptor (originally named GPR54, [1]) encoded by the *KISS1R* gene, with its cognate ligand, kisspeptin, in 2001 [2]–[4] led to a number of discoveries regarding the physiological functions of this system. Originally named metastin for its ability to inhibit the metastasis of some cancers [5], [6], kisspeptin has also been shown to be involved in trophoblast invasion during placentation [7]. However, recent interest has focused on kisspeptin as a crucial activator of the hypothalamic-pituitary-gonadal axis [8], [9]. Investigations in rats, mice, monkeys, sheep and humans have revealed that kisspeptin/kisspeptin receptor integrate internal and environmental cues to regulate the hypothalamic-pituitary-gonadal axis [10]–[21].

Kisspeptin, or KP-54, is a member of a larger group of peptides, the RF amides, named after their shared C-terminal amino acids [22]. RF amides have wide ranging roles in reproduction, feeding, blood pressure regulation and pain modulation with kisspeptins most closely implicated in reproduction and energy balance [21], [23]–[27]. KP-54 is cleaved from a single precursor, encoded by the *KISS1* gene, and smaller fragments of kisspeptin have been identified that are cleaved from KP-54 by unidentified proteases. These fragments, KP-14, KP-13 and KP-10, retain biological activity [3], suggesting that some or all of the C-terminal ten amino acids are essential for receptor activation. Cleavage of the three C-terminal amino acids of kisspeptin by the gelatinases MMP-2 and MMP-9 produces inactive fragments [28]. Additionally, kisspeptins are able to down-regulate MMPs, at both the transcriptional [29] and protein [30] levels. This may represent different levels/points of regulatory feedback under various circumstances, for instance in the different stages of pregnancy or in pathological states.

We have recently identified a further role for kisspeptin as a vasoactive peptide [31]. We detected kisspeptin and kisspeptin receptor expression in human vasculature, and intriguingly found this to be restricted to the aorta, coronary artery and umbilical vein, vessels with the same developmental origin. We also showed kisspeptin to be a potent vasoconstrictor in human coronary artery and umbilical vein [31], presumably via activation of the smooth muscle receptors. However, to our knowledge, a cardiac role of kisspeptin has not yet been investigated in man, nor any cardiovascular effects identified in other species. Our aim was therefore to compare the expression and pharmacological characteristics of the kisspeptin receptor in human, rat and mouse cardiac tissues. In human heart we have now demonstrated expression of mRNA encoding the kisspeptin receptor in cardiomyocytes and receptor protein was detected in human atrial and ventricular myocardium. Functionally, kisspeptins elicited potent inotropic activity in human paced atrial strips. Comparable receptor localisation and function were demonstrated in rat and mouse heart, and importantly loss of inotropic function of kisspeptins was observed in mice in which the *Kiss1r* gene is disrupted (*Kiss1r* ^−/−^), confirming a direct role for the kisspeptin receptor. A potential alteration in cardiac kisspeptin receptor signaling was indicated by a significant decrease in kisspeptin levels in the hearts of patients with ischaemic heart disease, and a trend to a corresponding compensatory increase in receptor density.

## Results

### Expression of mRNA encoding kisspeptin receptor in human cardiomyocytes

**Figure 1:**
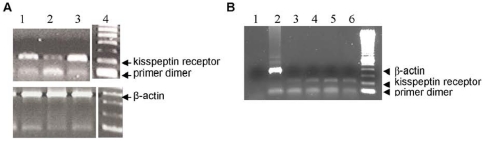
Detection of mRNA for kisspeptin receptor in human cardiomyocytes. (A) Expression of kisspeptin receptor mRNA in samples (n = 3) of human myometrium (Lanes 1–3), used as a control to confirm the specificity of PCR primers as this is a tissue in which the receptor protein has been previously identified. The integrity of the samples was confirmed by detection of the β-actin product of the expected size, 353 bp, indicating integrity of cDNA and absence of gDNA. PCR product size was determined using a 100 bp DNA ladder (Lane 4) (B) Expression of kisspeptin receptor mRNA (expected size 198 bp) in cDNA from human isolated cardiomyocyte samples (n = 3, lanes 3–5). β-Actin control (Lane 2) acted as a positive control and the absence of cDNA (Lane 1) served as a negative control with a 100 bp DNA ladder (Lane 6).

Integrity of the cDNA samples was established by the identification of bands of the expected size for products of the β-actin gene (353 bp) (Figure 1 A and 1B). The specificity of the kisspeptin receptor mRNA primers was confirmed by the presence of bands of the expected size (198 bp) in samples from human myometrium (Figure 1A), a tissue previously reported to express kisspeptin receptor protein [3]. Expression of kisspeptin receptor mRNA was identified in samples of human isolated cardiomyocytes (Figure 1B) confirming the potential of human heart tissue to express kisspeptin receptor protein.

### Immunohistochemical localisation of kisspeptin and kisspeptin receptor protein

**Figure 2:**
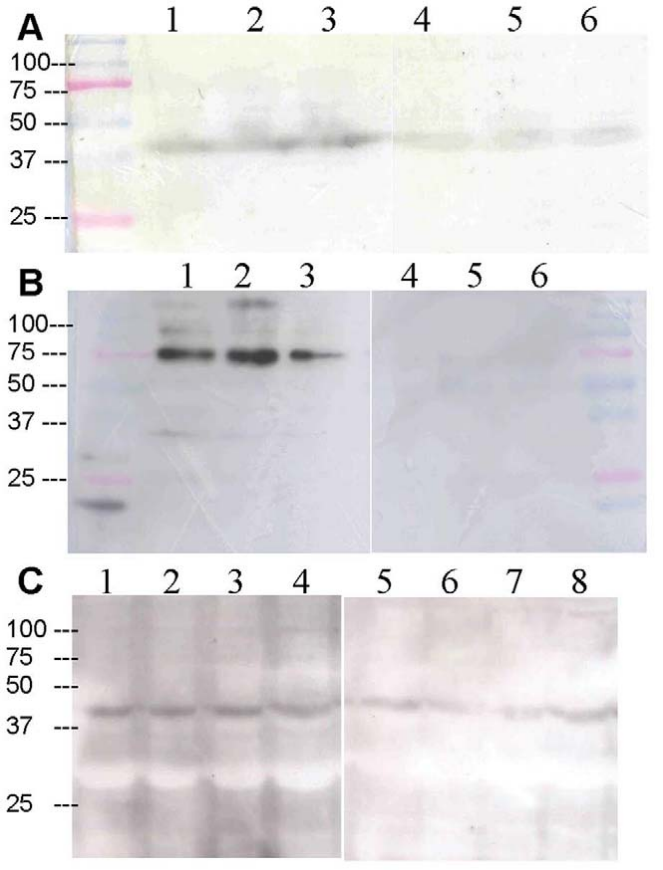
Detection of kisspeptin receptor protein in rat, mouse and human heart. Western blot analysis of rabbit anti-kisspeptin receptor (230–244, rat/mouse) serum on membrane preparations of (A) rat (n = 3) and (B) mouse (n = 3) heart revealed a single band of the expected size, 43 kDa or 75 kDa respectively (lanes 1–3). Pre-absorption of the primary antibody with the immunizing peptide resulted in attenuation of the bands (lanes 4–6). (C) Human left ventricle (n = 4) was probed with rabbit anti-kisspeptin receptor (375–398, human) (lanes 1–4) producing a band at 42 kDa, the expected size, which was attenuated when the primary antibody was pre-absorbed with the immunizing peptide (lanes 5–8). Protein marker positions are shown alongside the blots.

Western blotting revealed bands of the appropriate size for the kisspeptin receptor; rat 43 kDa (Figure 2A); mouse 75 kDa (Figure 2B); human 42 kDa (Figure 2C), that were consistent with previous reports [32]. In tissue from all three species, bands were attenuated by pre-absorption of the primary antibody with the appropriate immunizing peptide.

**Figure 3:**
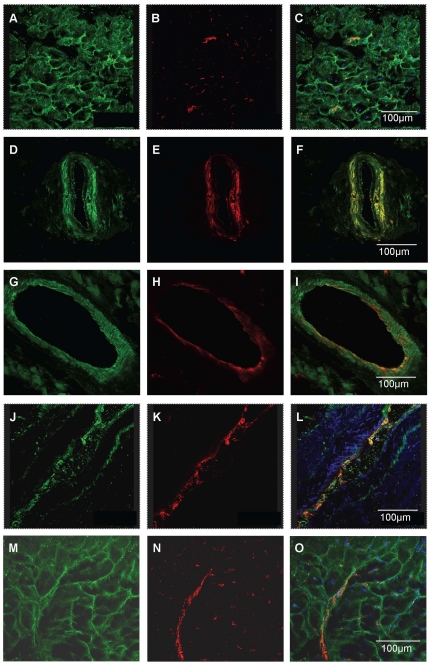
Cellular localisation of kisspeptin receptor-LI in human cardiovascular tissues. Representative photomicrographs showing kisspeptin receptor-LI localised to cardiomyocytes (A–C), co-localised with smooth muscle α-actin (red fluorescence E) to vascular smooth muscle of intramyocardial blood vessels (F) and with von-Willebrand factor (red fluorescence B, H) to vascular endothelial cells (A, I). Kisspeptin-LI (green fluorescence J, M) co-localised with von Willebrand factor (K, N) to vascular (L) and endocardial (O) endothelial cells and surrounding cardiomyocytes (O).

In sections of human heart kisspeptin receptor-like immunoreactivity (−LI) was detected in myocardium from all regions of explanted hearts investigated (right and left atria, right and left ventricle and interventricular septum) and in sections of atrial appendage obtained from coronary artery bypass graft operations. Within the myocardium, receptor protein localised to both cardiomyocytes (Figure 3 A–C) and intramyocardial blood vessels, with cell specific markers identifying localisation to both vascular smooth muscle (Figure 3 D–F) and endothelial cells (Figure 3 G–I). Kisspeptin-like immunoreactivity was detected in vascular (Figure 3 J–L) and endocardial endothelial cells in addition to cardiomyocytes (Figure 3 M–O).

**Figure 4:**
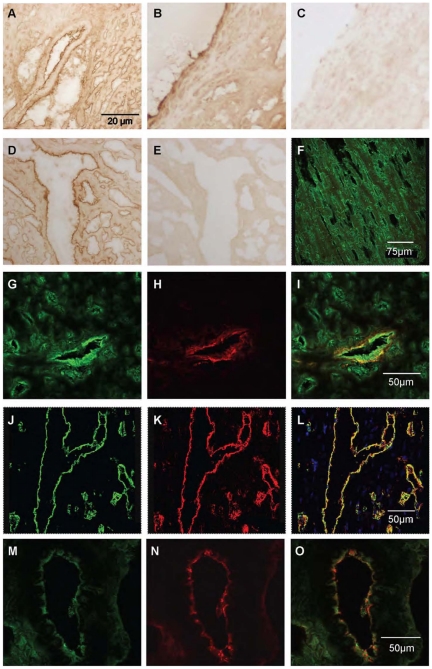
Localisation of kisspeptin receptor-LI in rat cardiovascular tissues. Representative photomicrographs showing peroxidase-antiperoxidase detection of kisspeptin receptor–LI to endothelial cells and underlying smooth muscle of (A) a small intramyocardial blood vessel, (B) large diameter thoracic aorta and (D) endothelial cells lining chambers of the heart. Pre-absorbed controls (C) and (F) obtained in adjacent tissue sections. Cellular localisation of kisspeptin receptor-LI was also identified as green fluorescence in (F) rat cardiomyocytes, (G) smooth muscle cells and (J) endothelial cells. Cell specific markers (H) smooth muscle α-actin and (K) von Willebrand factor are shown as red fluorescence and co-localisation with kisspeptin receptor confirmed by yellow fluorescence in the overlays (I, L). Kisspeptin-LI (M) localised to endothelial cells (O) identified by von Willebrand factor (N,) and to surrounding cardiomyocytes (M,O).

Similar expression patterns were obtained in rat cardiovascular tissues. Kisspeptin receptor-LI was present in endothelial cells and to a lesser extent to the underlying smooth muscle of small diameter intramyocardial blood vessels (Figure 4A) and large diameter thoracic aorta (Figure 4B) and to endocardial endothelial cells lining the chambers of the heart and surrounding myocytes (Figure 4D). Specificity of staining was confirmed by comparison to pre-immune controls (for example Figure 4C and E). Using confocal microscopy kisspeptin receptor-LI (green fluorescence) was identified in rat cardiomyocytes (Figure 4F) and co-localised with markers for both vascular smooth muscle cells (Figure 4 G–I) and endothelial cells (Figure 4 J–L). Kisspeptin-LI exhibited a more discrete localisation to endocardial and vascular endothelial cells with low expression in surrounding cardiomyocytes (Figure 4 M–O).

**Figure 5:**
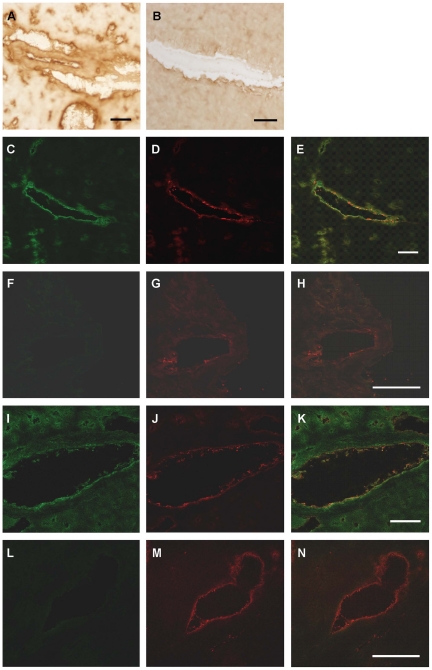
Localisation of kisspeptin receptor-LI in mouse heart. Representative photomicrographs showing peroxidase anti-peroxidase staining for (A) kisspeptin receptor-LI and (B) kisspeptin-LI in vascular and endocardial endothelial cells in mouse heart. In wild type mice kisspeptin receptor-LI (green fluorescence, C) and von-Willebrand factor-LI (red fluorescence D) co-localised (E) to vascular endothelial cells with receptor-LI also localised to adjacent cardiomyocytes whereas (F) no receptor-LI was detected in heart from *Kiss1r* ^−/−^ mice although endothelial cells could be visualized (G, H). Similarly, kisspeptin co-localised with von Willebrand factor in vascular endothelial cells (I–K) but was absent in heart tissue from *Kiss1* ^−/−^ mice (L–M). Scale bars are 50 µm.

In mouse heart kisspeptin receptor-LI localised to cardiomyocytes and intramyocardial blood vessels (Figure 5A), confirmed by dual labeling fluorescent microscopy (Figure 5C–E). Kisspeptin receptor–LI was not observed in *Kiss1r* ^−/−^ mice (Figure 5F–G). Similarly, kisspeptin-LI was present in small blood vessels and cardiomyocytes (Figure 5B) and co-localised with von Willebrand factor to endothelial cells lining the small blood vessels and chambers of the mouse heart (Figure 5 I–K) but was absent in sections of heart from *Kiss1* ^−/−^ mice (Figure 5L–N).

### [^125^I]KP-14 binding in human and rat heart

By receptor autoradiography specific binding of [^125^I]KP-14 was observed in all regions of human and rat myocardium. Additionally, specific binding was detected in the smooth muscle of human aorta, human small coronary resistance vessels and rat aorta. Receptor densities were similar for human myocardium from all regions (*P*<0.05, ANOVA), although higher levels were observed in aorta (Table 1).

**Table 1:** Autoradiographical analysis of [^125^I]KP-14 binding densities in human and rat cardiovascular tissues.

	Tissue	[^125^I]KP-14 specific binding (amol mm^−2^)	n
**Human**	Left atria	59±16	3
	Right atria	36±7	3
	Left ventricle	34±8	3
	Right ventricle	31±7	3
	Interventricular septum	29±10	3
	Apex	27±6	4
	Atrial appendage	51±15	4
	Small resistance vessel	4±3	5
	Aorta	214±46	6
**Rat**	Heart	29±10	5
	Aorta	21±2	3

Data are expressed as mean ± SEM. n-Values are the number of individuals/animals from which tissue was obtained.

In saturation experiments [^125^I]KP-14 bound monophasically in human right atria. Binding was saturable with subnanomolar affinity (K_D_ 0.12±0.05 nM, B_MAX_ 8±5 fmol mg^−1^ protein, n = 3). In rat heart [^125^I]KP-14 also bound with a comparable subnanomolar affinity and receptor density (K_D_ 0.44±0.14 nM, B_MAX_ 11±2 fmol mg^−1^, n = 6). In both species Hill slopes were close to unity suggesting presence of only one binding site, or multiple sites with the same affinity.

### Inotropic activity in atrial tissue

**Figure 6:**
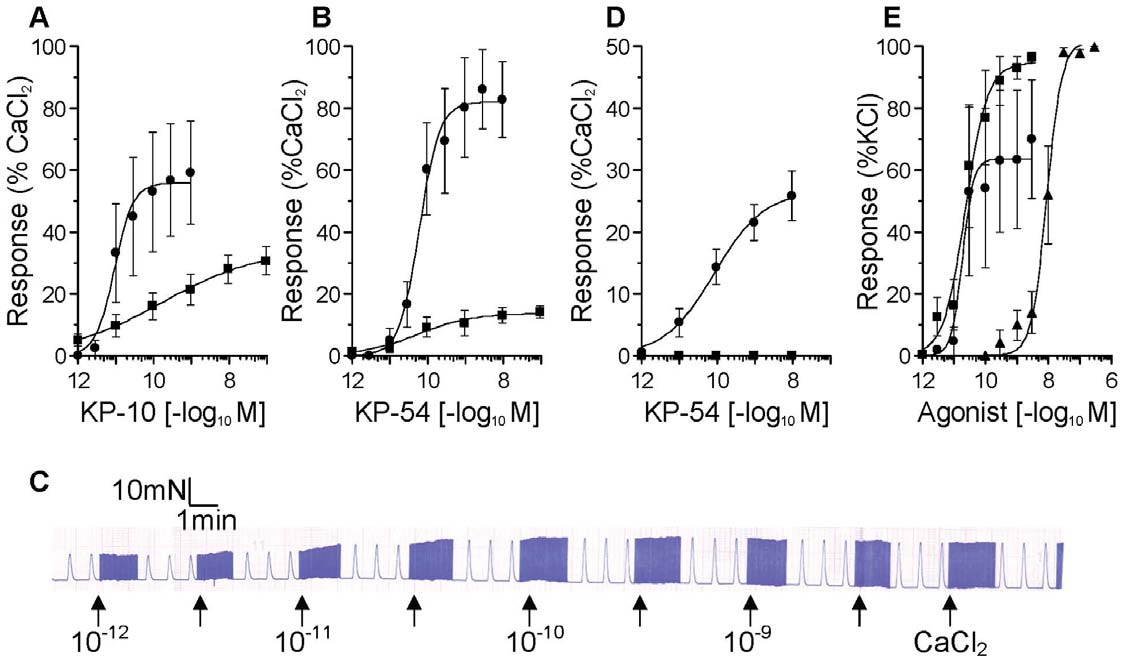
Functional responses to kisspeptins in human, rat and mouse cardiovascular tissues. Concentration-response curves showing positive inotropic effects of (A) KP-10 and (B) KP-54 on strips of human atrial appendage (•) and rat atria (▪). An example of the chart recorder trace for a single human atrial strip is shown (C). The chart recorder speed was increased before the addition of each concentration of kisspeptin to allow analysis of lusitropic responses (half time to peak force and half time to relaxation) for individual peaks. (D) Concentration-response curves showing the positive inotropic effect of KP-54 on *Kiss1r* ^+/+^ mouse atria (•) and lack of effect on *Kiss1r* ^−/−^ atria (▪). (E) Vasoconstrictor responses to KP-10 (•), KP-54 (▪) and ET-1 (▴) in endothelium-denuded rat aortic rings. All values are mean ±SEM.

In human paced atrial strips and rat paced paired atria there was no significant difference in the mean baseline contractile force generated at 50% of optimum resting tension or in mean contractile force in response to 8.95 mM CaCl_2_ between tissues used to test KP-10 and KP-54. In human atria both peptides were positive inotropes with comparable sun-nanomolar potency (KP-10 pD_2_ 9.99±0.73, n = 5; KP-54 pD_2_ 10.39±0.44, n = 5) and maximum responses (KP-10 E_MAX_ 59±10%, KP-54 E_MAX_ 82±13%) (Figure 6 A, B).

Inotropic responses in rat paired atria were of similar potency but with lower maximum response than for human atria (KP-10 pD_2_ 9.70±0.44, E_MAX_ 31±5%, n = 7; KP-54 pD_2_ 9.85±0.63, E_MAX_ 14±2%, n = 4) (Figure 6 A, B). Lusitropic effects were not observed in either species to either peptide (Figure 6C).

In mouse atria, positive inotropic effects of KP-54 were observed, with comparable potency (pD_2_ 10.21±0.34, n = 6) to those in human and rat and maximum response (E_max_ 26±4%) similar to that obtained in rat atria. No response to kisspeptin was detected in mice with targeted disruption of *Kiss1r* (Figure 6D).

### Vasoconstrictor activity in endothelium-denuded rat aorta

Aortic rings from all rats tested contracted to ET-1 (pD_2_ 8.13±0.11, E_MAX_ 99±1%, n = 7). Responses were more variable for the kisspeptins, with vasoconstrictor responses obtained to KP-10 (pD_2_ 9.96±0.53, E_MAX_ 76±17% KCl) in tissues from 4 animals and KP-54 in tissues from 3 animals (pD_2_ 10.47±0.26, E_MAX_ 97±1% KCl) (Figure 6 E).

### KISS1 in human heart disease

**Figure 7:**
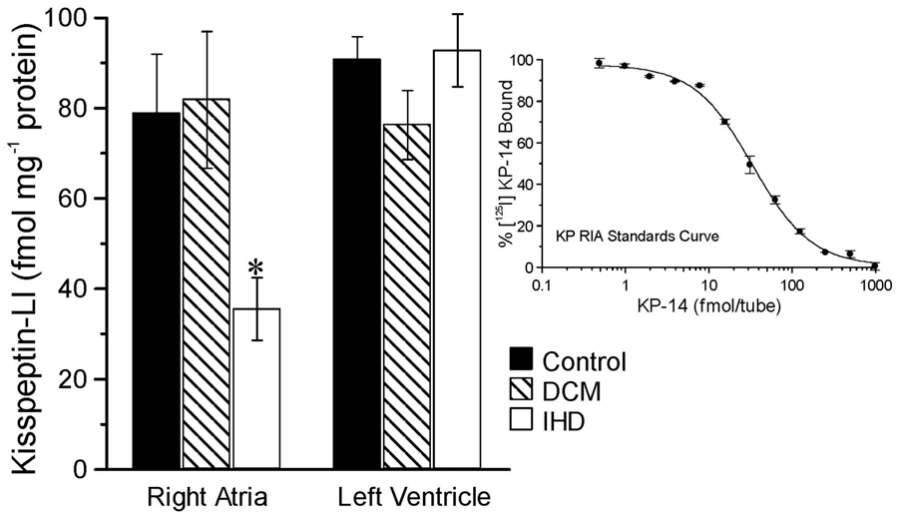
Detection of kisspeptin-LI in human heart. Levels of kisspeptin-LI detected in human right atria and left ventricle compared from donor hearts (control), hearts from patients transplanted for dilated cardiomyopathy (DCM) or ischaemic heart disease (IHD). Values are mean ± SEM. For right atria levels of kisspeptin-LI were significantly decreased compared to the other two groups (*P*<0.05, ANOVA). Inset graph is an example of a standards curve.

The level of kisspeptin-LI detected in myocardium of right atria from control hearts (n = 4) was not different from that in myocardium from patients transplanted for dilated cardiomyopathy but there was a significant reduction in myocardium from right atria of patients transplanted for ischaemic heart disease (*P*<0.05, ANOVA). In left ventricle, there was no significant difference in kisspeptin-LI between the three groups (*P*>0.05) (Figure 7).

To determine if the change in peptide level detected in the right atria was associated with an alteration in receptor density, saturation analysis was performed with [^125^I]KP-14. No significant difference in receptor density or binding affinity in right atria from control, dilated cardiomyopathy (DCM) or ischaemic heart disease (IHD) patients was observed (Table 2).

**Table 2:** Saturation binding analysis of [^125^I]KP-14 in sections of human normal and diseased right atria.

	K_D_ (nM)	B_MAX_ (fmol mg^−1^ protein)	nH	n
**Control**	0.12±0.05	8.0±4.8	0.82±0.10	3
**DCM**	0.11±0.06	10.2±3.4	1.00±0.02	3
**IHD**	0.46±0.17	13.5±7.0	0.94±0.03	3

K_D_: the concentration of [^125^I]KP-14 which at equilibrium occupies 50% of the binding sites; B_MAX_: receptor density; nH: Hill coefficient; DCM: dilated cardiomyopathy; IHD: ischaemic heart disease. Values are mean ± SEM.

## Discussion

The RF-amide peptide family, which also includes prolactin releasing peptide, are known to have roles in cardiovascular regulation, both centrally [33] and peripherally [34], [35]. However, to date mRNA studies of kisspeptin receptor distribution in whole heart samples have reported low but varying mRNA expression by northern blot [2], expression was not detected by PCR [3], [4], [32] except by Sawyer and colleagues, who were also able to detect mRNA encoding murine kisspeptin, albeit at very low copy number [46]. We now report for the first time, the expression of kisspeptin and the kisspeptin receptor protein in human, rat and mouse heart, and have shown that kisspeptins are potent positive inotropes in *in vitro* cardiac preparations from these species. Phylogenetic analysis has revealed that the kisspeptin receptor is most closely related to the galanin receptors (GAL_1–3_). Galanin receptors are not thought to mediate major cardiovascular actions, however, there is evidence that galanin can modulate cholinergic transmission, inhibiting parasympathetic slowing of the heart following activation of the cardiac sympathetic system [36] for example after myocardial infarction [37]. The urotensin II receptor (UT) shows some sequence similarity to both kisspeptin and galanin receptors and urotensin-II has been shown to be a vasoactive peptide [38] and a potent inotrope in human heart [39].

Consistent with the inotropic actions of the kisspeptins we have shown that kisspeptin receptor localises to cardiomyocytes in sections of human, rat and mouse heart. Furthermore, we have confirmed receptor protein expression in human and rat heart by receptor autoradiography with saturation binding analysis revealing comparable subnanomolar affinity in both species for the radioligand [^125^I]KP-14, comparable to that previously reported in human blood vessel smooth muscle [31] and cell lines expressing the receptor [3], [4]. The receptor densities that we measured were similar to other inotropic peptides in native human tissue such as apelin (B_MAX_ 3.1 fmol mg^−1^, [40]). We found no evidence for more than one receptor subtype, as predicted from the genome, in contrast to non-mammalian species where two subtypes of kisspeptin receptor have been cloned, *Kiss1r* and *Kiss2r* [41].

The creation of mice with targeted disruption of the *Kiss1r* gene [18] has permitted further investigations into the inotropic effects of kisspeptins. In atria from wild-type mice, KP-54 was a potent positive inotropic agent, with a similar potency to that obtained in rat and human tissues and a similar maximum response to that observed in rat. The differences in the E_MAX_ values for both KP-10 and KP-54 that we observed between human and rodent may reflect altered responsiveness of the rodent receptor to the human peptides used in these experiments. KP-10 differs by one amino acid: the C-terminal residue is phenylalanine in human and tyrosine in rat and mouse. The full-length kisspeptin sequence, KP-54, contains 22 amino acid differences. Significantly, no inotropic response to KP-54 was detected in the *Kiss1r* ^−/−^ mice, confirming that the inotropic effects of kisspeptin are mediated solely by this receptor, at least in this species.

Circulating levels of kisspeptin are low, (0.83±0.19 pM, [31]) and so we were interested to identify a local cardiac source of kisspeptin. In all three species we detected kisspeptin-LI both to cardiomyocytes and to endocardial endothelial cells lining the chambers of the heart. Whether this endogenous cardiac kisspeptin system has a significant physiological role remains to be determined, however we have some evidence that levels of kisspeptin are altered in ischaemic heart disease with a significant reduction of peptide expression in right atria. This may not be unexpected; in the failing heart, both MMP-2 and MMP-9 are up-regulated [42] and, importantly, these enzymes cleave kisspeptins to biologically inactive products [28] that would not be detected by our radioimmunoassay. Changes in endogenous ligand concentration for GPCRs can result in changes in expression levels of receptor protein and we did detect a trend to increased receptor density in the right atria of diseased hearts which was not significant, but may be a consequence of downregulation of peptide.

Our discovery that kisspeptins are positive inotropic agents in human and rodent heart is the first report of such activity. These inotropic effects were very potent and comparable to other peptides such as endothelin (pD_2_ = 8.9) [43] and apelin (pD_2_ = 9.9) [44]. A number of existing studies have shown that the kisspeptin receptor is coupled to G_q/11_, leading to phospholipase C activation and Ca^2+^ release [3], [4], [32] and that Rho and Rho-associated kinase are activated by KP-10 [45]. Activation of these signaling pathways would be consistent with function as both an inotropic agent and a vasoconstrictor. We have previously shown that in the human vasculature expression of kisspeptin receptor in large diameter human vessels was restricted to vessels with a common developmental origin, umbilical vein, coronary artery and aorta, the latter prone to development of atherosclerosis [31]. In the present study we have confirmed that kisspeptin receptor protein is expressed on both the vascular smooth muscle and endothelial cells of small intramyocardial vessels in the hearts of all three species in addition to these cell types in the rat aorta. Functional experiments confirmed that activation of the smooth muscle receptor in rat aorta mediated vasoconstriction. Although responses were variable, in those tissues that did contract the kisspeptins were more potent than ET-1. In a recent study Sawyer and colleagues [46] have demonstrated that injected KP-10 attenuated microvascular cutaneous blood flow in mice, suggesting that kisspeptins may have a more generalized constrictor potential on resistance blood vessels compared to larger vessels. Consistent with kisspeptins acting as paracrine mediators we localized kisspeptin-LI to vascular endothelial cells lining the small intramyocardial vessels in human, rat and mouse heart. KP-54 was originally identified as a metastasis suppressor protein [5] and subsequently shown to be anti-angiogenic [47]. Locally mediated vasoconstriction may be one mechanism by which kisspeptins modify blood flow to tumors and limit their metastatic potential.

Humans with mutations in *KISS1R* develop hypogonadotropic hypogonadism, characterised by decreased levels of sex steroids and delayed puberty [9]. However, no cardiovascular phenotype has been observed in these patients. The same is also true in mice with targeted disruption of either *Kiss1r* or *Kiss1*, suggesting that the kisspeptin system is not crucial for normal cardiovascular physiology. In healthy individuals, circulating kisspeptin levels are very low, perhaps reflecting, as suggested for the heart, local rather than endocrine actions. However, plasma kisspeptin concentrations are markedly increased 10000-fold above normal levels in pregnancy [48], and we can speculate that under these conditions kisspeptin may contribute to the adaptive increase in cardiac output [49]. There is also evidence that kisspeptins contribute to the pathogenesis of pre-eclampsia. Kiss1 mRNA has been reported to be increased in pre-eclampsia patients [50] and if kisspeptins have a general vasoconstrictor action in the microvasculature then this increase may contribute to the hypertensive phenotype in these individuals. However, a conflicting report detected decreased Kiss1 mRNA levels [51] in pre-eclampsia, suggesting this is an area that warrants further investigation.

In conclusion, we have detected the expression of kisspeptin and kisspeptin receptor in human, rat and mouse myocardium and vasculature and have shown kisspeptins to be potent positive inotropes in the atria of these three species. The (patho)physiological relevance of these data remains to be determined. However, small molecular weight kisspeptin receptor agonists may have promise as novel therapies for attenuation of metastasis in tumors or for triggering puberty in individuals with delayed onset. Therefore, it is important to recognize at an early stage of the development of such compounds their potential for cardiovascular actions.

## Materials and Methods

### Ethics Statement

Anonymised human cardiovascular tissue samples were used in this study with local ethical approval from the Huntingdon Research Ethics Committee (REC 05/Q0104/142). The samples were obtained from Papworth Hospital Research Tissue Bank (Cambridgeshire 1 Research Ethics Committee reference 08/H0304/56, samples collected with written informed patient consent). Transgenic mice and wild type litter mates were bred and maintained in the combined animal facility, Department of Physiology, Development and Neuroscience, Cambridge University, under Home Office project licence number PPL 80/2143. This project was subject to approval by a Local Ethical Committee at the University of Cambridge before submission to the Home Office. Animals were maintained on a normal diet with free access to water and food, in a climatically controlled environment and were killed by CO_2_ exposure in accordance with Schedule 1 of the U.K. Animals (Scientific Procedures) Act, 1986.

### Tissues

Human cardiovascular tissues were from 40 male and 12 female patients, age range 17–78 years. Ventricular and atrial myocardium, apex and interventricular septum were from the explanted hearts of patients transplanted for dilated cardiomyopathy, ischaemic heart disease or from control hearts. Atrial appendages were obtained from patients receiving coronary artery bypass grafts. Rat tissues were obtained from male Sprague-Dawley (340–450 g) and Wistar (215–280 g) rats (Charles River Laboratories, Margate, UK). Mouse tissues were obtained from adult C57/Bl6/J (20–30 g) male mice and 129S6/SvEv wild type and transgenic (*Kiss1*r^−/−^ (Gpr54*^tm1PTL^* [9]) and *Kiss1* ^−/−^ (Kiss1*^tm1PTL^* [18]) male mice (2–4 months old).

### Myocyte isolation, RNA extraction and Reverse Transcription

Human cardiomyocytes were isolated as previously described [52]. mRNA was extracted from myometrium and cardiomyocytes using a single step guanidinium isothiocyanate method. cDNA synthesis was carried out using Invitrogen SuperScript III First-Strand Synthesis System for RT-PCR and oligo-dT primer. To confirm the integrity of the cDNA samples PCR reactions were performed using primers spanning an intronic region in the β-actin gene: 5′ primer 5′-GCTCGTCGTCGACAACGGCT-3′; 3′ primer 5′CAAACATGATCTGGGTCATCTTCTC.

Conditions were 98°C for 1 minute, followed by 40 cycles of 97°C for 1 minute, 55°C for 30 seconds, 72°C for 1 minute, and a final extension at 72°C for 10 minutes. Expected product size was 353 bp.

Primers for kisspeptin receptor were designed using Invitrogen oligoperfect ™ designer: 5′ primer 5′-CTCGCTGGTCATCTACGTCA-3′; 3′ primer, 5′-CCAGTTGTAGTTCGGCAGGT-3′.

Optimized PCR conditions were 98°C for 1 minute, followed by 30 cycles of 97°C for 1 minute, 59°C for 30 seconds, 72°C for 1 minute, and a final extension at 72°C for 10 minutes. Expected product size 198 bp.

The reaction mixtures consisted of 10× PCR buffer, 0.2 mM dNTPs, 1.5 mM MgCl_2_, 0.2 µM of each primer, and 1 U of Platinum® Taq DNA polymerase. PCR products were separated by electrophoresis on a 1% agarose gel with ethidium bromide and sizes estimated by reference to a 100-bp DNA molecular weight standard. Specificity of the kisspeptin receptor primers was confirmed using mRNA extracted from human myometrium a tissue known to express the receptor [3]. Negative controls in which cDNA was omitted were included for each PCR reaction.

### Protein Detection

To examine the expression of kisspeptin receptor protein in rat, mouse and human heart homogenates, Western blotting was performed using species specific anti-kisspeptin receptor antibodies raised against amino acids 230–244 of the rat/mouse receptor sequence and 375–398 of the human receptor. Briefly, rat and mouse hearts and human left ventricular tissue were homogenized in lysis buffer (mM; Tris, 50; MgCl_2_, 5; EDTA, 5; EGTA, 1; protease inhibitor cocktail (mM: 4-(2-aminoethyl)benzenesulphonyl fluoride, 104; bestatin, 4; leupeptin, 2; pepstatin A, 1.5; E-64, 1.4; aprotonin, 0.08), 1∶1000; pH 7.5 at 22°C), filtered through gauze and centrifuged at 30 000 g for 30 minutes at 4°C. Pellets were re-suspended in 1 ml lysis buffer and re-centrifuged. The resulting pellet was suspended in 500 µl HEPES/KCl buffer (mM; HEPES, 50; KCl, 13; protease inhibitor cocktail, 1∶1000; pH 8.0, 22°C) and protein concentration determined using the Biorad DC 96-well microtitre plate system. Protein samples were reduced and denatured by heating at 95°C in Laemelli sample buffer (mM: 10% SDS; Glycerol; Tris,1; 1% bromophenol blue; Dithiothreitol (DTT), 1) and electrophoresed on a 10% SDS-polyacrylamide gel with molecular weight markers. Samples were transferred to a polyvinylidene fluoride membrane using a semi-dry blotting method and blocked in Tris-buffered saline (TBS) (mM: Tris, 0.41) with 5% non-fat dry milk overnight at 4°C. Membranes were probed with 1∶1000 of the appropriate rabbit anti-kisspeptin receptor serum in TBS/0.5% Tween-20 (TBS/T) and 1% non-fat dry milk overnight at 4°C before incubation for 1 h at 22°C with 1∶5000 goat anti-rabbit horseradish peroxidase (Amersham, Bucks, U.K.) in TBS/T with 1% non-fat dry milk. Controls were performed in which the rabbit anti-kisspeptin receptor sera were incubated with the immunizing peptides (1 µM) overnight, at 4°C (pre-absorbed control), before probing the membrane; or the primary antibody was omitted from the protocol (negative control). Detection was performed with enhanced chemiluminescence (ECL) reagent and exposure to Kodak film.

### Immunohistochemistry

Immunohistochemistry (IHC) was carried out using standard techniques [53]. Cryostat-cut sections (10 µm) of rat, mouse and human heart were fixed in acetone for 10 minutes at 4°C prior, blocked (to reduce non-specific staining) in PBS for 2 hours at 22°C with either 5% swine serum (SS) for peroxidase anti-peroxidase IHC or, for dual-labeling fluorescent IHC, in either 5% goat serum (GS) for human and rat tissues or 5% donkey serum for mouse tissues. Human right atria, rat heart and mouse heart sections were incubated with the appropriate species specific anti-kisspeptin receptor or anti-kisspeptin sera. For dual-labeling fluorescence IHC sections were also incubated with mouse (human and rat tissues) or sheep (mouse tissues) anti-von Willebrand Factor (vWF) or mouse anti-smooth muscle alpha actin (SMαA). Controls were performed by incubating adjacent sections with either primary antibody pre-absorbed with the immunizing peptide or pre-immune sera if available or the primary antibody was omitted.

For peroxidase anti-peroxidase IHC, immunoreactivity was visualized by incubation with swine anti-rabbit secondary antibody and reaction with 3,3′-diaminobenzidine (DAB) and examined using a bright-field microscope (Olympus UK LTD, Essex, UK). Images were captured using a U-TV1-X digital camera (Olympus UK LTD, Essex, UK) and CellD software. For dual-labeling fluorescence IHC sections were incubated with the species appropriate AlexaFluor-488 and AlexaFluor-568 conjugated secondary antibodies before visualization via laser scanning confocal microscopy (Leica Microsystems, Bucks, UK).

### Fluorescence Dual Labeling Immunocytochemistry (ICC)

Sections (10 µm) of human heart were fixed in acetone and blocked with 5% non-immunized goat serum (GS) in phosphate buffered saline (PBS). Sections were co-incubated with 1∶100 rabbit anti-GPR54 (375–398, human) serum or rabbit anti-KP-10 (KP(45–54)-NH_2_, human) and 1∶50 mouse anti-vWF monoclonal antibody. Secondary antibody solution contained 1∶200 AlexaFluor 488 conjugated goat anti-rabbit serum and AlexaFluor 568 conjugated goat anti-mouse serum. Visualization was carried out using laser scanning confocal microscopy [31].

### Radioligand binding

For receptor autoradiography, cryostat-cut sections of human (15 µm) or rat (10 µm) heart were pre-incubated with phosphate binding buffer (mM: NaH_2_PO_4_, 50; NaCl, 100; EDTA, 5; MgCl_2_, 5; protease inhibitor cocktail, 1; pH 7.4) before incubation for 2 h with 0.2 nM [^125^I]KP-14 [31]. Unlabelled KP-14 (10 µM) (human tissues) or KP-10 (1 µM) (rat tissues) defined non-specific binding in adjacent sections. Equilibrium was broken by washing in 50 mM Tris-HCl buffer, pH 7.4, 4°C before air-drying and apposition to radiation-sensitive film (Kodak Biomax MR) and determination of specific binding by computer-assisted densitometry using the Quantimet 970 system (Leica Microsystems, Bucks, UK).

For saturation analysis, sections were incubated with increasing concentrations of [^125^I]KP-14 (20 pM–500 nM for human tissues, 4 pM–2 nM for rat tissues) with 1 µM unlabelled KP-14 or KP-10 included to define non-specific binding in adjacent sections [54]. [^125^I]KP-14 binding was detected using a gamma counter to measure disintegrations per minute (dpm) and total protein content was calculated using the Biorad 96-well microtiter plate system.

### In vitro pharmacology

Paired rat or mouse atria, or 4 mm strips of human atrial appendage, were mounted in 10 ml organ baths containing oxygenated Krebs solution at 37°C as described previously [55]. Tension on the tissue was adjusted to 50% of that at which the maximum force was generated and then paced at 1 Hz, square-wave pulse, 5 ms duration, with the voltage just above threshold (<4.0 V). The isometric tension generated to KP-10 and KP-54 (1 pM–300 nM) was recorded and concentration-response curves constructed. Experiments were terminated by the addition of 6.7 mM CaCl_2_ (final bath concentration 8.95 mM) and responses expressed as a % of this CaCl_2_ maximum.

Rat thoracic aorta was cleaned of connective tissue and cut into 4 mm rings and the endothelial layer removed by gentle rubbing. Tissues were set to a basal tension at which the maximum response to 100 mM KCl was generated and subsequent responses to kisspeptins were expressed as % of this KCl response [38]. Aortic rings were then contracted with 10 µM phenylephrine and 1 µM ACh was added; a reduction in the phenylephrine response of <10% was taken as evidence of removal of endothelium. Cumulative concentration-response curves were constructed to KP-10, KP-54 (10^−12^ M–3×10^−9^ M) and as a control endothelin-1 (ET-1, 10^−10^ M–3×10^−7^ M). Experiments were terminated by the addition of 100 mM KCl and agonist responses were expressed as a % of this KCl maximum.

### Radioimmunoassay

Pooled human plasma (n = 4, male) was acidified and used for characterization of the radioimmunoassay (RIA). Samples of human left and right atria, left and right ventricle, apex and interventricular septum were homogenized, boiled for 15 min in 0.5 M acetic acid and spun at 20000 g for 30 min at 4°C. Peptides were extracted using silica based columns, dried and reconstituted in radioimmunoassay (RIA) buffer (mM: Na_2_HPO_4_, 40; NaH_2_PO_4_, 10; Na Azide, 8 and gelatin, 0.025%; Tween 20, 0.05%, pH 7.4 at 22°C).

RIA was carried out using a previously characterised assay [31], with inter- and intra-assay variation <10%, a detection limit of 2.8 fmol/tube, an IC_50_ of 34 fmol/tube and the linear portion of the curve ranging from 8–200 fmol/tube. Incubates consisted of 100 µl of standard (0–10 nM KP-14) or extracted sample, 100 µl rabbit anti-KP-10 (KP(45–54)-NH_2_, human) (1∶30000) and 100 µl [^125^I]KP-14 (10000–15000 cpm), diluted in RIA buffer. Free and bound kisspeptin was separated by addition of activated charcoal and [^125^I]KP-14 detected using a gamma counter.

### Statistical Analysis

Radioligand binding study data were analyzed using the iterative, non-linear curve fitting programs EBDA and LIGAND in the KELL package (Biosoft, Cambridge, UK). Pooled K_D_, B_MAX_ and Hill slopes (nH) were expressed as mean±SEM and normalized to protein concentrations. *In vitro* pharmacology data agonist responses were expressed as %CaCl_2_ response in the paced atrial tissues and %KCl in the vasoconstrictor studies and data were fitted to a four parameter logistic equation using an iterative curve-fitting program (FigSys, Biosoft, Cambridge, UK) to obtain values for potency (pD_2_: the negative logarithm to the base 10 of the EC_50_) and maximum response (E_MAX_: %CaCl_2_ or %KCl, respectively) expressed as mean±SEM. Where appropriate, values were compared by one-way analysis of variance (ANOVA). In all cases *P*<0.05 was considered statistically significant and n-values were the number of animals or patients from which data were obtained.

### Drugs and Reagents

KP-54, KP-14 and KP-10 (rat/mouse) peptides were custom synthesised by Severn Biotech (Kidderminster, UK). KP-10 (human) and ET-1 (human) were from Peptide Institute (Osaka, Japan). [^125^I]KP-14 (2000 Ci/mmol) was synthesised by Amersham Biosciences (GE Healthcare, Little Chalfont, UK), prepared from unlabelled KP-14 by direct iodination with sodium [^125^]-iodide using the chloramine-T method. Rabbit anti-KISS1 (raised against amino acids 375–398 of the human receptor sequence) serum and rabbit anti-KP-10 (KP(45–54)-NH_2_, human) serum were from Phoenix Pharmaceuticals (Belmont, CA, USA). Rabbit anti-Kiss1 serum, raised again amino acids 230–244 of the rat/mouse KISS1 sequence, was custom synthesised by Severn Biotech (Kidderminster, UK) and rabbit anti-KP-10 (rat/mouse) was kindly provided by Dr. Alain Caraty (Unité de Physiologie de la Reproduction et des Comportements, Institut National de la Recherche Agronomique/Centre National de la Recherche Scientifique/Université Tours, Nouzilly, France) [56]. Swine anti-rabbit secondary antibody, mouse anti-vWF and mouse anti-SMαA were from from Dako UK (Ely, Cambridgeshire, UK) and sheep anti-vWF was from Abcam (Cambridge, UK). AlexaFluor488-conjugated goat anti-rabbit, AlexaFluor568-conjugated goat anti-mouse, AlexaFluor488-conjugated donkey anti-rabbit and AlexaFluor568-conjugated donkey anti-sheep secondary antibodies were from Molecular Probes (Leiden, The Netherlands). All molecular biology reagents, including primers, were from Invitrogen (Paisley, UK), all other reagents from Sigma-Aldrich (Poole, UK).
